# Single-Cell Glia and Neuron Gene Expression in the Central Amygdala in Opioid Withdrawal Suggests Inflammation With Correlated Gut Dysbiosis

**DOI:** 10.3389/fnins.2019.00665

**Published:** 2019-07-03

**Authors:** Sean J. O’Sullivan, Evangelia Malahias, James Park, Ankita Srivastava, Beverly A. S. Reyes, Jon Gorky, Rajanikanth Vadigepalli, Elisabeth J. Van Bockstaele, James S. Schwaber

**Affiliations:** ^1^Daniel Baugh Institute for Functional Genomics and Computational Biology, Department of Pathology, Anatomy and Cell Biology, Thomas Jefferson University, Philadelphia, PA, United States; ^2^Department of Chemical Engineering, University of Delaware, Newark, DE, United States; ^3^Institute for Systems Biology, Seattle, WA, United States; ^4^Department of Pharmacology & Physiology, Drexel University College of Medicine, Philadelphia, PA, United States

**Keywords:** opioid dependence, addiction, withdrawal, inflammation, amygdala, single-cell gene expression, microflora

## Abstract

Drug-seeking in opioid dependence is due in part to the severe negative emotion associated with the withdrawal syndrome. It is well-established that negative emotional states emerge from activity in the amygdala. More recently, gut microflora have been shown to contribute substantially to such emotions. We measured gene expression in single glia and neurons gathered from the amygdala using laser capture microdissection and simultaneously measured gut microflora in morphine-dependent and withdrawn rats to investigate drivers of negative emotion in opioid withdrawal. We found that neuroinflammatory genes, notably *Tnf*, were upregulated in the withdrawal condition and that astrocytes, in particular, were highly active. We also observe a decreased *Firmicutes* to *Bacteroides* ratio in opioid withdrawal indicating gut dysbiosis. We speculate that these inflammatory and gut microflora changes contribute to the negative emotion experienced in opioid withdrawal that motivates dependence.

## Introduction

Opioid dependence has grown at an alarming rate over the past decade. Rehabilitation services are overwhelmed (Sigmon, 2014) and maintenance therapies have proven their value and limitations (Stotts et al., 2009). These circumstances motivate investigation into non-canonical mechanisms of addiction pathophysiology to identify novel treatment targets.

Inflammation has been shown to play a role in drug dependence (Coller and Hutchinson, 2012). Recently, opioids have been shown to increase central cytokine and chemokine production, and the mechanisms explaining this phenomenon are coming to light (Peterson et al., 1998; Wang et al., 2012; Jacobsen et al., 2016). Moreover, human trials suggest that anti-inflammatory pharmacotherapies may be efficacious in treating opioid withdrawal symptoms (Cooper et al., 2016). Opioid-induced neuroinflammation may contribute to analgesic tolerance (Watkins et al., 2009) and dependence (Evans and Cahill, 2016). These findings have spurred investigation into the effect of opioids on microglia and astrocytes: the principle neuroimmune regulators in the central nervous system (CNS).

Areas of the CNS involved in addiction have demonstrated differing immunologic responses to both opioid exposure and withdrawal (Hutchinson et al., 2009). Herein, we focus on the central nucleus of the amygdala (CeA) building on our previous work demonstrating inflammation in the CeA in alcohol withdrawal (Freeman et al., 2012a, b, 2013). The amygdala, and CeA in particular, is strongly implicated in opioid dependence (Koob, 2009a; Upadhyay et al., 2010; Lyons et al., 2013) and is thought to be principally responsible for the negative emotion experienced in opioid withdrawal (Koob, 2009b). Inflammation in the amygdala has been shown to cause anxiety-like behavior (Yang et al., 2016). The negative reinforcement theory of addiction postulates that avoidance of these negative emotions, and corresponding physical symptoms, motivates opioid dependence (Baker et al., 2004; Koob, 2009a; Evans and Cahill, 2016). Additionally, the amygdala is a hub in the interoceptive vagal circuit (Figure 9) that responds to peripheral inflammation and gut dysbiosis—both of which have been linked to anxiety (Critchley and Harrison, 2013; Holzer et al., 2015; Maniscalco and Rinaman, 2018).

We gathered single neurons, microglia, and astrocytes from the CeA of control, opioid-dependent, and naltrexone-induced withdrawn rats using laser-capture microdissection (LCM) (Espina et al., 2006). We measured a subset of the transcriptional profiles of these single cells in 10-cell pools with a microfluidic quantitative (q)PCR platform Biomark (Fluidigm^TM^). Transcriptional profiles from morphine-dependent animals did not differ substantially from control animals, but withdrawn animals differed from both. Astrocytes demonstrated the most profound changes with many of the genes assayed upregulated and strongly correlated in the withdrawal condition. Strikingly, tumor necrosis factor-alpha (*Tnf*) was significantly upregulated in each cell type in withdrawal consistent with neuroinflammation (*p* < 0.05 nested ANOVA). Increased TNF-α protein was found with Western blot and immunofluorescence. Additionally, we assayed gut microflora in opioid dependence, naltrexone vehicle, and withdrawal and observed a lowered *Firmicutes* to *Bacteroides* ratio in withdrawal indicating dysbiosis.

## Materials and Methods

### Animals

Two cohorts of adult male Sprague-Dawley rats (230–250 g), *n* = 12 and 16, respectively, ordered from Harlan Laboratories (Indianapolis, IN) were housed in 12 h light, 12 h dark cycles at 20 C° and given free access to food. Animals were randomly divided into three or four treatments: (1) Placebo, (2) Morphine, (3) Naltrexone, and (4) Withdrawal. All animals underwent subcutaneous placement of two slow-release pellets at hour 0. Pellets in Placebo and Naltrexone animals contained no drug. Morphine and Withdrawal animals received insertion of two slow-release pellets containing 75 mg each of morphine base (National Institute of Drug Abuse). Placebo and Morphine animals were sacrificed by rapid decapitation at 144 h (6 d) following pellet insertion producing moderate morphine dependence (Koob et al., 1992). Naltrexone and Withdrawal animals were given an intraperitoneal injection of naltrexone (100 mg/Kg) at hour 144 (6 d) and sacrificed 24 h later (hour 168; 7 d). Timing and dosage were chosen based on previous experiments demonstrating morphine dependence and withdrawal following similar protocols (Rasmussen et al., 1990; Scavone and Van Bockstaele, 2009). Cohort 1 (12 animals) was used for single-cell gene expression, Western blot, and first cecal sample analysis and did not include Naltrexone treatment. Cohort 2 (16 animals) was used for immunofluorescence imaging and second cecal sample analysis.

### Rapid Decapitation, Fast Staining Protocol, LCM

Brief isofluorane anesthesia was followed by rapid decapitation and immediate collection of brain and cecal samples. Forebrains were frozen into Optimal Cutting Temperature (O.C.T.) for cryostat sectioning while cecal samples were placed in conical tubes. All samples were frozen at −80°C to preserve nucleic acid integrity. A rapid immunofluorescent staining protocol developed in-house to reduce nucleic acid degradation was used to label cell types for single-cell laser capture microdissection (LCM) selection as explained elsewhere (Park et al., 2016). Briefly, 10 μm thick sections were thaw-mounted onto glass slides and exposed to 30 s of 75% ethanol to fix sliced tissue followed by 30 s of 2% BSA (Sigma-Aldrich) in phosphate-buffered saline (PBS) for blocking. Fixed tissue was stained with 3% of anti-NeuN antibody (EMD Millipore, MAB377), anti-Cd11β antibody (Genway Biotech, CCEC48), or anti-GFAP antibody (Thermo Fischer, A-21294) as a primary stain to label cell types. Slices were then washed with PBS containing 2% BSA and incubated with secondary antibody Alexa-488 anti-mouse (1:200 ratio) and DAPI (1:10000). Following a final PBS wash, slides underwent a standard alcohol dehydration protocol (30 s 75% ethanol, 30 s 95% ethanol, 30 s 100% ethanol, 30 s 100% ethanol, 60 s xylenes, 4 m xylenes) and were transferred to a desiccator for 5 m before LCM was employed (Espina et al., 2006).

### Cecal Material Collection and Analysis

Two cohorts of 12 and 16 rats were studied for these experiments. As a pilot, the first cohort of 12—from which single brain cells were collected—had their cecal material analyzed by microfluidic qPCR with the aid of the Microbial DNA qPCR Array Intestinal Infections kit (Catalog#: 330261). DNA was extracted with the QIAamp Fast DNA Stool Mini Kit (Catalog#: 51604). The same kit was used for DNA extraction of cecal samples from the 16 rat cohort. qPCR primers to measure cecal bacterial abundance in second cohort were designed in-house based on literature (Supplementary Table S7).

### Single Cell Sampling and High-Throughput qRT-PCT

Single brain cells, 1060 neurons, 1070 microglia, and 1060 astrocytes, were collected from the central nucleus of the amygdala using LCM as 10-cell pooled samples. Reverse transcription generated cDNA from mRNA transcripts (SuperScript^TM^ VILO^TM^ cDNA Synthesis Kit; ThermoFisher). cDNA was then pre-amplified (22 cycles) with 96 pairs (forward and reverse) of PCR primers using TaqMan PreAmp Master Mix. Expression levels of 96 genes were measured using a high-throughput quantitative PCR platform (Biomark, Fluidigm©). Probe-based qPCR measured the levels of the previously amplified 96 cDNA primer. 96.96 dynamic gene expression arrays were employed. A list of probe and primer sets is included in Supplementary Table S1. Agarose gel electrophoresis was used to validate primer amplicons.

### Data Normalization and Analysis

We normalized expression levels using a two-step −ΔΔC_t_ method (Spurgeon et al., 2008). Briefly, expression of a gene within a single sample was measured as a raw *C*_T_ value. These values were normalized to the geometric mean of the most stable housekeeping genes (*Ldha* and *Actb* for brain and Pan Bacteria or Universal primer controls for gut) within that sample. Housekeeping gene stability was determined by both expression variance and geNorm (Vandesompele et al., 2002). This yields −ΔC_t_ values which were then median-centered across all samples for that gene providing a −ΔΔC_t_ value for each sample allowing comparison of relative gene expression values across treatment groups and batches. All data normalization and analysis was performed using the R (v3.2.3) programming language. Differential expression statistics were calculated using nested ANOVA (*n* = 4 animals for each treatment). Differential abundance of microflora statistics were calculated using two-way ANOVA. Pearson correlation coefficients were calculated with the Harrell Miscellaneous (Hmisc) R package. Correlations meeting a *q* < 0.001 are displayed as edges and thickness is based on absolute value of the Pearson correlation coefficient (strength of correlation). Gene correlation networks were constructed using Cytoscape^®^ version 3.7.1.

### Western Blot

200 μm punches from the CeA were removed from flash-frozen forebrain hemisections (Left). Tissue homogenization occurred in RIPA lysis buffer (1% phenylmethylsulfonyl fluoride (PMSF), 1% sodium orthovanadate and 2% protease inhibitor). Pierce BCA Protein Assay Kit (23227, Life Technologies, Carlsbad) was used for protein estimation of lysate. An equal amount of protein was run on 12% SDS-PAGE (Mini-protean, Bio-Rad, Richmond, CA, United States), blocked with 5% Blotting-Grade Blocker (1706404, Bio-Rad, Richmond, CA, United States) and incubated with anti-TNF-α antibody (Abcam, ab9755; 1:500) overnight at 4°C. TBST [25 mM Tris (pH 7.60), 137 mMNaCl, and 0.1% Tween 20] was used to wash the membrane. HRP-conjugated secondary antibody was used to probe the membrane. Immunoreactive bands were visualized on Kodak Image Station 440CF by chemiluminescent Clarity (TM) Western ECL Substrate (1705060, Bio-Rad, Richmond, CA, United States).

### Immunofluorescence Staining and Confocal Microscopy

Frozen forebrains sectioned at 20 μm thickness with cryostat were thaw mounted on glass slides. CeA and occipital lobe regions were fixed in 4% paraformaldehyde (Electron Microscopy Sciences, Hatfield, PA, United States) and rinsed in PBS three times, 5 min each. Sections were permeabilized with 0.02% Triton X-100 (LabChem, Zelienople, PA, United States) for 15 min. Following two 5 min PBS washes, sections were blocked with 5% bovine serum albumin (ab7481, Cambridge, MA, United States) (BSA) PBS for 1 hour. Overnight incubation with primary antibody occurred at 4°C. Primary antibody included anti-NeuN antibody (EMD Millipore, MAB377), anti-Cd11β antibody (Genway Biotech, CCEC48), or anti-GFAP antibody (Thermo Fischer, A-21294) (1:100), and anti-TNF-α (Abcam, ab9755; 1:100). Afterward, slides were washed 3 times for 5 min each and incubated in the dark with the secondary antibody (Goat anti-mouse IgG Alexa Fluor 488; 1:500) for 1 h and 45 min at room temperature. Following three 5 min PBS washes, DAPI (D9542, Sigma) was applied and allowed to incubate for 15 min. Lastly, slides were washed 3 times for 5 min, mounted with ProLong Diamond Antifade (Life Technologies, Carlsbad, CA, United States), and stored in darkness at 4°C. Negative controls were imaged in absence of the primary antibody (data not shown).

Confocal microscopy using Zeiss LSM 780 mounted on a Zeiss axio observer inverted microscope was performed with the Zeiss ZEN 2011 software. We used lasers 405 nm (DAPI), 488 nm (cell type), 555 nm (TNF-α), and for image acquisition. Images were acquired at 1024 × 1024 pixel resolution, 8-bit color depth, and an average intensity of 4 line scans of the same area.

## Results

**FIGURE 1:**
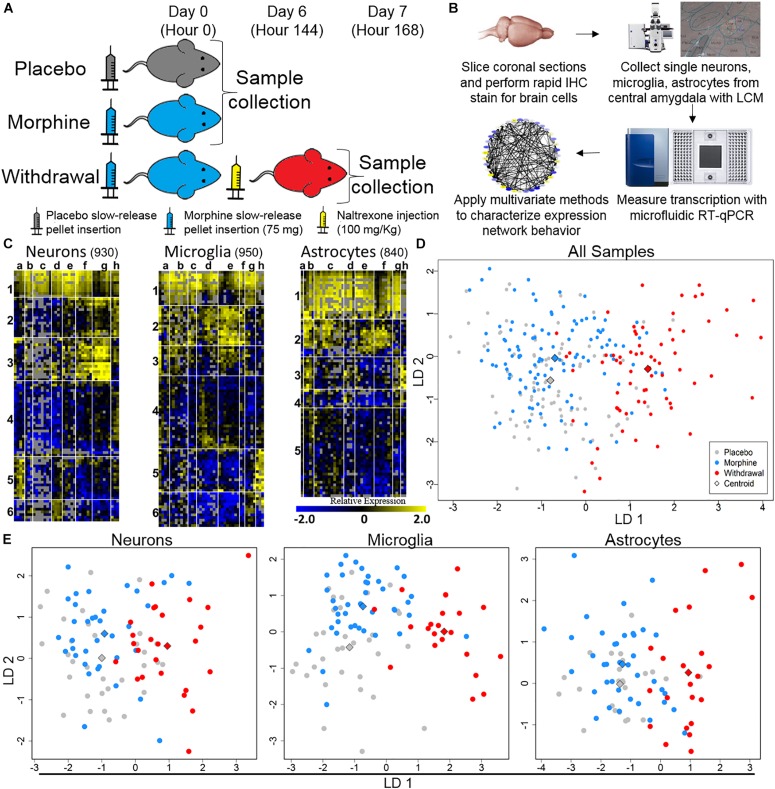
Single-cell RT-qPCR workflow and transcriptional heterogeneity. **(A)** Experimental protocol (*n* = 4 for each condition). **(B)** Single-cell transcriptome data generation. **(C)** Heat map shows expression of all samples across 40 assayed genes (see Supplementary Table S1 for gene primers). Rows are samples (10-cell pools; numbers denote sample clusters) and columns are genes (letters are gene clusters for that cell type; see Supplementary Table S2). Color denotes z-score of −ΔΔCt expression value representing relative gene expression. **(D)** Linear discriminate analysis (LDA) of all samples. Circles are 10-cell pooled samples and diamonds are centroids. A distance of 0.533 was found between Placebo and Morphine centroid points, 2.22 between Placebo and Withdrawal centroid points, and 2.12 between Morphine and Withdrawal centroid points (see Supplementary Table S3 for centroid analysis). **(E)** LDA of specific cell types, circles are 10-cell pooled and diamonds are centroids. In neurons, a distance of 0.594 was found between Placebo and Morphine centroids, 1.97 between Placebo and Withdrawal centroids, and 1.89 between Morphine and Withdrawal centroids. In microglia, a distance of 1.20 was found between Placebo and Morphine centroids, 3.01 between Placebo and Withdrawal centroids, and 2.65 between Morphine and Withdrawal centroids. In astrocytes, a distance of 0.485 was found between Placebo and Morphine centroids, 2.31 between Placebo and Withdrawal centroids, and 2.23 between Morphine and Withdrawal centroids.

We gathered 1060 neurons, 1070 microglia, and 1060 astrocytes from the CeA of rats that were either given placebo pellets (Placebo, *n* = 4), morphine pellets (Morphine, *n* = 4), or experienced 24 h of acute naltrexone-precipitated morphine withdrawal (Withdrawal, *n* = 4) (Figure 1A). 33,088 individual PCR reactions occurred using the microfluidic BioMark^TM^ platform (Fluidigm©) (Figures 1A,B). Strict quality control was employed to limit inaccuracies producing a dataset containing 13,650 individual data points. In total, the expression of 46 gene transcripts across 930 neurons, 950 microglia, and 840 astrocytes were analyzed (Figure 1C). Transcripts of proteins involved in inter and intracellular signaling, metabolism, oxidative stress, and inflammation were measured. Expression levels were corrected for differences in RNA input using the geometric mean of *Actb* and *Ldha*: the most stable and least variable housekeeping genes assayed (geNorm). *Gapdh* expression levels served as an independent control. Transcript measures that failed in any one batch due to poor signal quality or assay contamination were excluded from the entire dataset to ensure robust analysis of every gene included. Single-cell collection was validated by expression of the cell type markers *NeuN*, *Maf*, and *Gfap* (Supplementary Figure S1).

Dimension reduction analyses of this dataset were used to determine overall shifts in gene expression between treatments. This subset of the transcriptome was not markedly altered following 6 days of moderate morphine exposure. Linear discriminate analysis (LDA) yielded a distance of 0.533 between Placebo and Morphine centroid points (Supplementary Table S3 and Figure 1D). However, global expression was altered in the Withdrawal condition (LDA returned a distance of 2.22 and 2.12 between centroid points of Placebo and Withdrawal samples and Morphine and Withdrawal samples, respectively; Supplementary Table S3 and Figure 1D). This pattern persisted when samples were grouped by cell type (Supplementary Table S3 and Figure 1E). Astrocytes demonstrated the largest relative alteration of expression in opioid withdrawal (Supplementary Table S3).

Principle components (PC) 2 and 3 showed the clearest clustering of the data by treatments (Supplementary Figures S2A–C). A composite weight score for each gene ($C\,o\,m\,p\,o\,s\,i\,t\,e\,W\,e\,i\,g\,h\,t=\sqrt{\left(P\,C\,2^{2}+P\,C\,3^{2}\right)}$) is listed in Supplementary Table S4. Notably, *Tnf* and *Gfap* were among the most heavily weighted genes for each cell type and in Withdrawal. The endogenous opioid precursor gene *Pdyn* also contributed substantially to the observed separations.

**FIGURE 2:**
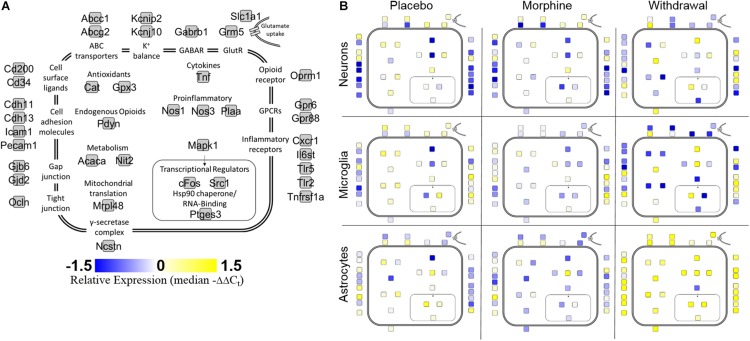
Cell diagram of gene expression changes across treatments and cell types. Colored squares represent relative gene expression (median −ΔΔCt value for gene denoted in panel **(A)**. Location of squares represents cellular localization or function of the corresponding protein. **(A)** Legend. **(B)** Panels display relative gene expression represented by color across treatments and cell types. Yellow is high expression, blue is low expression, white is neutral expression.

Cell diagrams (Figure 2) display relative gene expression (median of −ΔΔC_t_ values) denoted by color organized by gene function or protein location across the three treatments within each cell type. Neurons increased expression of transcriptional regulators and inflammatory receptors in Withdrawal while microglia decreased expression of antioxidant genes. Genes assayed in astrocytes mostly demonstrated increased expression in Withdrawal. Interestingly, *Mapk1*, an important regulator of substance dependence physiology (Reyes-Gibby et al., 2015), had decreased expression in withdrawal in all three cell types.

**FIGURE 3:**
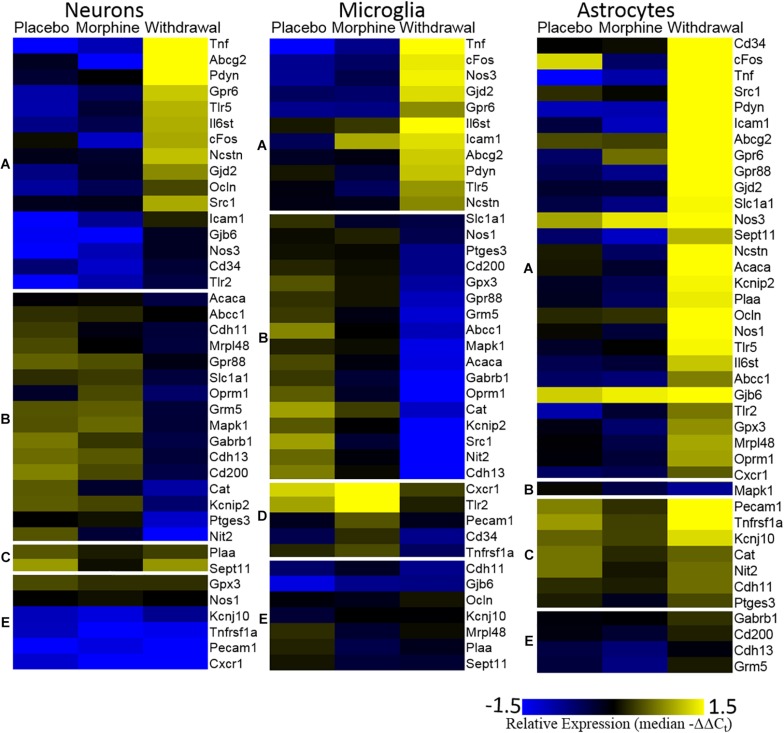
Heat map. Color represents relative gene expression (median −ΔΔC_t_ values) for labeled gene. Genes are clustered based on expression behavior across treatments. Gene clusters are denoted on the left as lower-case letters [**(A)** withdrawal induced; **(B)** withdrawal suppressed; **(C)** withdrawal rebound; **(D)** morphine induced; and **(E)** no change).

The Figure 3 heat map also plots relative expression by median −ΔΔC_t_ values within a gene with genes clustered by expression patterns. Generally, Placebo and Morphine samples had similar median expression values and genes were either induced or suppressed in Withdrawal. Of note, *Tnf*, *cFos*, and *Nos3* were upregulated in Withdrawal in all three cell types. Strikingly, astrocytes markedly upregulated most genes assayed suggesting that astroglia play a principle role in the neurochemical processes of opioid withdrawal in the CeA. We also note that expression of a group genes in microglia, including the inflammatory receptors *Cxcr1*, *Tlr2*, and *Tnfrsf1a*, was induced in Morphine but not Withdrawal. Similarly, *Tnf* expression in microglia was induced in Morphine and then more so by Withdrawal. Microglia are thought to be the only cell type in the CNS that express the opioid-responsive toll-like receptor 4 (TLR4) (Wang et al., 2012). This may explain why morphine-induced gene expression was only observed in microglia. This finding was unexpected and further suggests that astroglia drive the neuroinflammation observed in opioid withdrawal.

**FIGURE 4:**
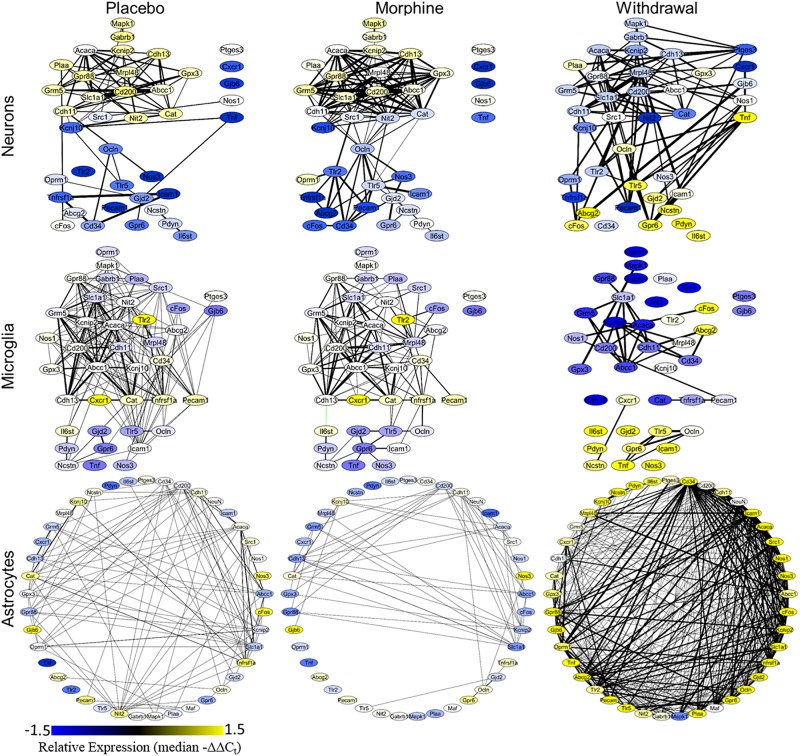
Gene correlation networks. Pearson correlation was performed on the −ΔΔC_t_ values within a treatment and cell type. Nodes denote genes and their color signifies relative expression levels (median −ΔΔC_t_ value for each gene). Edges denote expression correlations; thickness signifies strength of correlation (the absolute value of the Pearson correlation coefficient). Correlations that met a *q* < 0.001 cut off are displayed. Black edges are positive correlations and green edges are negative correlations. Gene correlation networks were constructed using Cytoscape^®^ version 3.7.1.

A Pearson correlation coefficient analysis was used to construct gene expression correlation networks to visualize gene expression correlation (Figure 4). Edge numbers and differences in edge numbers are listed in Supplementary Table S5. *Nos1*, *Ptges3*, and *Tnf* have zero correlations with the other genes assayed in the neuronal Morphine network. In the neuronal Withdrawal network, these genes are highly connected suggesting their expression is dysregulated in opioid withdrawal syndrome (Supplementary Table S5). In microglia, *Tnf* also demonstrated an increase in edge number from Morphine to Withdrawal conditions further implicating TNF-α signaling in the CeA in opioid withdrawal syndrome. In neurons and microglia, we also observe a general inversion of expression from Placebo and Morphine to Withdrawal in the two gene clusters (top vs. bottom) apparent in the Placebo and Morphine networks. This dichotomy suggests that the regulation of gene expression in Withdrawal is under a substantially different set of constraints than the homeostatic state in Placebo or allostatic state of Morphine. This may be an indicator of the importance of the withdrawal process itself in driving a pathologic state in the amygdala and contributing to dependence.

Astrocyte networks tell a different story. The total number of gene correlations (edges) increased substantially in Withdrawal (Supplementary Table S5). This is consistent with the above findings suggesting CeA astrocytes are highly active in opioid withdrawal. Further investigation into the role of astroglial in the CeA in opioid dependence and withdrawal is required, but based on these findings, we conclude that astrocytes are central to altered glial-neuronal signaling and inflammation in this process.

**FIGURE 5:**
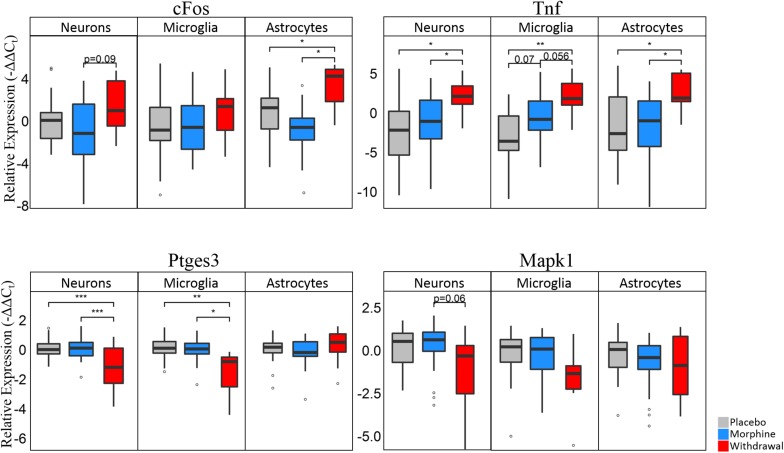
Boxplots of select genes demonstrating significant differential gene expression. Statistics were calculated using nested ANOVA (^*^p < 0.05, ^∗∗^*p* < 0.01, ^∗∗∗^*p* < 0.0001, *n* = 4 animals for all treatments). See Supplementary Figure S3 for bar plots showing individual samples. See Supplementary Table S6 for all nested ANOVA *p*-values.

Differential gene expression statistics (nested ANOVA) can be found in Supplementary Table S6. Boxplots of expression levels of *cFos*, *Tnf*, *Ptges3*, and *Mapk1* are displayed in Figure 5 (see Supplementary Figure S3 for plot with data points). Astrocytes have a significant increase in *cFos* expression in Withdrawal (nested ANOVA, *n* = 4 animals, ^*^p < 0.05) further supporting their activation in opioid withdrawal. Increased *cFos* expression was found in withdrawal neurons as well, but not significant (*p* = 0.09). Every cell type had a significant increase in *Tnf* expression in Withdrawal with microglia showing an almost significant increase (*p* = 0.07) from Placebo to Morphine. Increased CeA TNF-α protein in Withdrawal was validated by Western blot analysis and immunofluorescence (IF) (Supplementary Figures S4–S6). Western blot and IF imaging confirmed that TNF-α was present at low levels in Morphine and higher levels in Withdrawal. IF imaging showed TNF-α signal in the extended amygdala region only. Surrounding regions and the occipital lobe which was assayed as a negative control (not shown) did not show TNF-α IF. Cell-type staining did not strongly implicate a specific cell type responsible for TNF-α but TNF-α staining was mostly concentrated around neurons.

**FIGURE 6:**
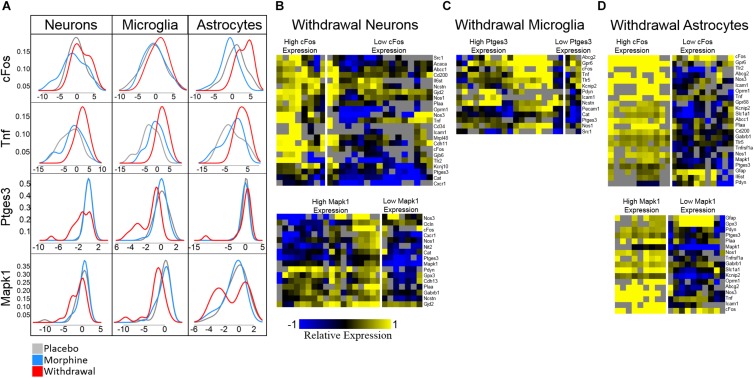
Density plots and subphenotype heat maps. Color denotes relative expression and is derived from −ΔC_t_t values normalized to the mean of Placebo expression. **(A)** Density plots show bimodal expression distribution among withdrawal samples for some genes in some cell types. **(B)** Heat maps comparing subphenotypes of Withdrawal neurons. **(C)** Heat map comparing subphenotype of Withdrawal microglia. **(D)** Heat maps comparing subphenotypes of Withdrawal astrocytes.

*Ptges3* demonstrated the strongest differential expression of all genes assayed. This transcript codes for the p23 protein which acts a cochaperone protein with heat shock protein 90 (Hsp90) (Felts and Toft, 2003), is a prostaglandin E2 synthesis enzyme (Tanioka et al., 2000), and functions as an RNA binding protein in macrophages (Liepelt et al., 2016). We found it substantially downregulated in neurons and microglia in opioid Withdrawal. Heat shock proteins are known to be differentially expressed in opioid dependence and withdrawal but the functional significance of low Ptges3 expression is unknown (Ammon et al., 2003; Rodriguez Parkitna et al., 2004). *Mapk1* (*Erk2*) expression was also decreased in Withdrawal in neurons (*p* = 0.06) and microglia but not significantly. We show *Mapk1* here as the best example of a gene that demonstrated bimodal expression by astrocyte subphenotypes in Withdrawal as seen in Figure 6A density plots.

Density plots for *Mapk1* expression in neurons and astrocytes reveal that *Mapk1* expression segregates into two groups of Withdrawal samples: high and low expressing groups (Figures 6B,D). We observe the same phenomenon in *cFos* expression in neurons and astrocytes and in *Ptges3* expression in microglia (Figures 6B–D). Figures 6B–D compares a subset of the expression profile of high-*cFos* expressing Withdrawal neurons to low *cFos-*expressing Withdrawal neurons. Notably, low-expressing *cFos* Withdrawal neurons had very low *Oprm1* expression which may account for their non-reactivity in opioid withdrawal indicated by low *cFos*. In astrocytes, high *cFos*-expressing Withdrawal samples demonstrate a phenotype characterized by a general increase in expression of the genes displayed (Figure 6B) further suggesting these astrocytes in particular are highly active. This subphenotype analysis illuminates the importance of singe-cell studies and the cellular subphenotypes that may be missed by tissue-level sampling.

**FIGURE 7:**
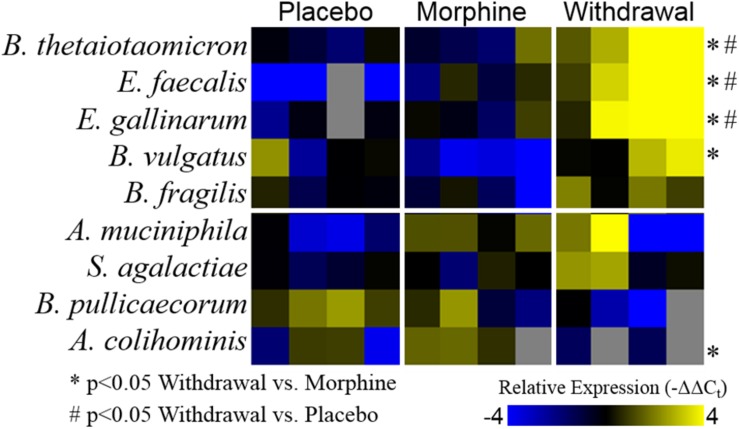
Relative abundance of gut microflora from rat cohort 1. Heat map displays relative abundance of bacterial species (−ΔΔC_t_ values). Rows are bacterial species and columns are samples from different rats. ^*^p < 0.05 Withdrawal compared to Morphine, #*p* < 0.05 Withdrawal compared to Placebo; two-way ANOVA *n* = 4 animals for each treatment.

**FIGURE 8:**
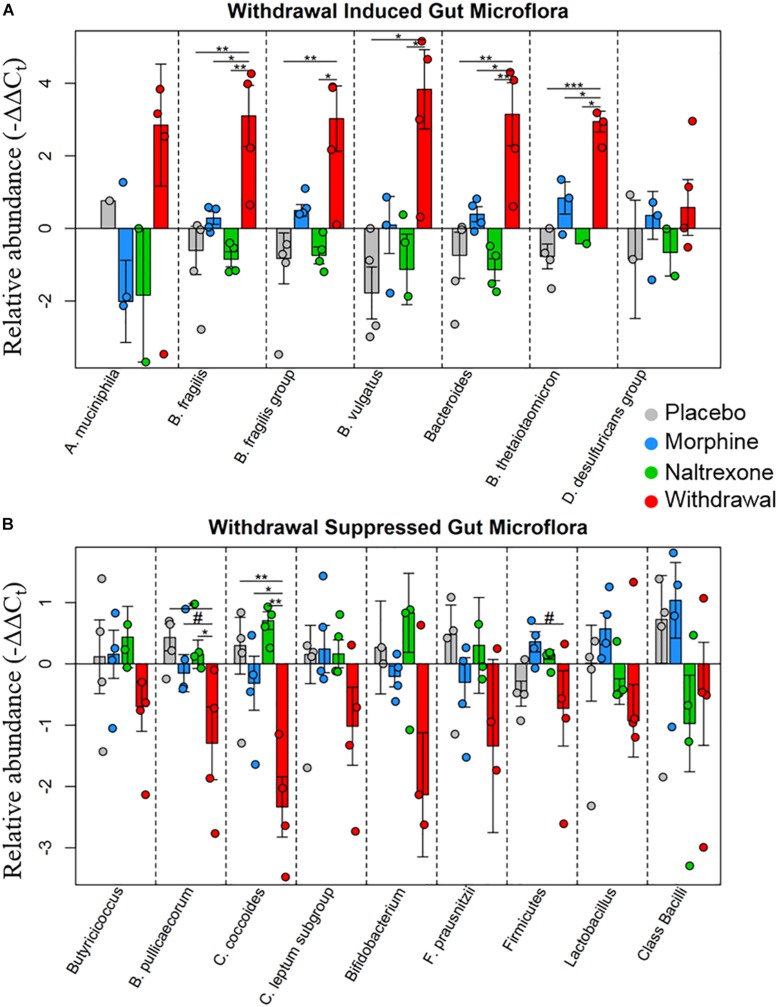
Relative abundance of gut microflora from rat cohort 2. Barplots display relative abundance of bacterial species (−ΔΔC_t_ values). Error bars are standard error. #*p* < 0.1, ^*^p < 0.05, ^∗∗^*p* < 0.008, ^∗∗∗^*p* = 0.0009; two-way ANOVA *n* = 4 animals for each treatment. **(A)** Withdrawal induced microbes. **(B)** Withdrawal suppressed microbes.

**FIGURE 9:**
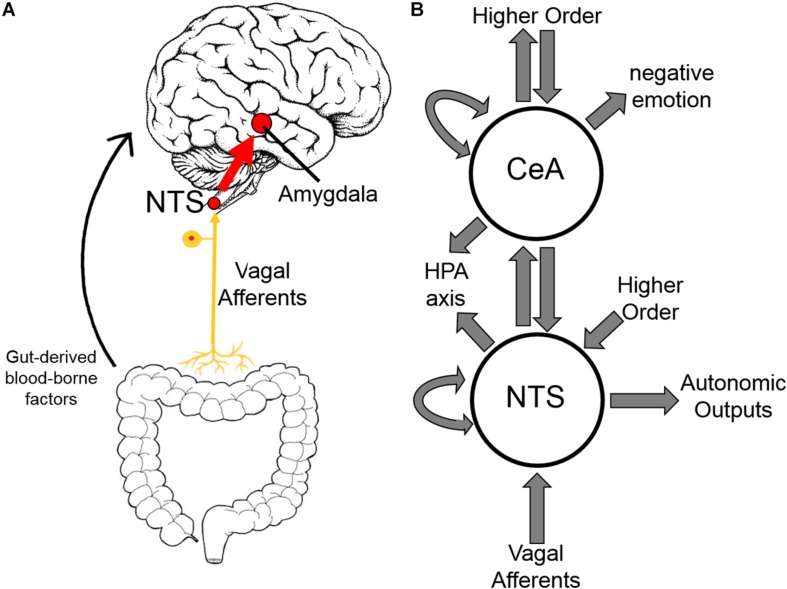
Interoceptive vagal circuit and visceral-emotional neuraxis. **(A)** Interoceptive vagal afferents relay the state of the gut to the nucleus tractus solitarius (NTS). This information is subsequently relayed to the amygdala and influences emotional states. Gut-derived blood-borne factors are shown as another important aspect of the gut-brain connection. **(B)** A simplified cartoon representation showing the integrative roles of the nucleus tractus solitarius (NTS) and the central nucleus of the amygdala (CeA) in emotion, stress, and autonomic regulation. Many anatomical and functional connections are omitted for clarity (HPA axis, hypothalamic-pituitary-adrenal axis).

Based on the emerging evidence of the influence of gut microflora on emotions and behavior, we measured the relative abundance (qPCR) of gut microbiota in both rat cohorts. The first set of rats showed significant upregulation of species *Bacteroides thetaiotaomicron*, *Enterococcus faecalis*, *Enterococcus gallinarum*, and *Bacteroides vulgatus* species in opioid withdrawal (Figure 7). Interestingly, *E. faecalis* and *B. vulgatus* are associated with inflammatory bowel disease while *B. thetaiotaomicron* is thought to be anti-inflammatory (Kelly et al., 2004; Scott et al., 2013). We confirmed these preliminary findings with the second cohort of rats and further identified bacterial phyla, class, genii, and species that were induced or suppressed by opioid withdrawal (Figure 8 and Supplementary Table S8). The two major phyla that comprise mammalian gut microflora, *Firmicutes* and *Bacteroides*, shifted in opposite directions lowering the *Firmicutes* to *Bacteroides* ratio (Placebo = 1:0.75, Morphine = 1:1.03, Naltrexone = 1:0.42, Withdrawal = 1:4.67; Table 1) in Withdrawal which is an established marker of inflammation and dysbiosis (Tamboli et al., 2004; Collins, 2014; Sampson et al., 2016; Rowin et al., 2017). The subgroups of these phyla shifted in the same direction as the phyla validating this finding. The *Bifidobacterium* genus and *Faecalibacterium prausnitzii*, which also have established anti-inflammatory properties, were also suppressed in Withdrawal (Figure 8; Sokol et al., 2008; O’Callaghan and van Sinderen, 2016). These alterations in gut microfloral abundance may influence the observed gene expression and inflammatory changes in the amygdala via the interoceptive vagal circuit (Figure 9).

**TABLE 1:** *Firmicutes* to *Bacteroides* ratio across all treatments.

	**Placebo**	**Morphine**	**Naltrexone**	**Withdrawal**
*Firmicutes* (F) median −ΔΔCt value	−0.487	0.359	0.151	−0.726
*Bacteroides* (B) median −ΔΔCt value	−0.741	0.391	−0.424	2.943
Normalization factor (NF)	1.487	0.641	0.849	1.726
(F+NF) : (B+NF)	1 : 0.75	1 : 1.03	1 : 0.42	1 : 4.67

## Discussion

The central nucleus of the amygdala (CeA) is a limbic hub involved in autonomic regulation, emotion, and motivated behavior, and has been strongly implicated in opioid dependence (Koob, 2009a; Upadhyay et al., 2010). We found that 6 days of chronic moderate morphine exposure does not substantially influence the transcriptional state of single neurons, microglia, and astrocytes in the CeA but that morphine withdrawal does. Strikingly, astrocytes demonstrated the most profound shift in the measured subset of the transcriptome. In addition, significant upregulation of the proinflammatory cytokine *Tnf* was observed in opioid withdrawal in all three cell types assayed suggesting local paracrine signaling in the CeA during opioid withdrawal is shifted toward a neuroinflammatory state. Increased TNF-α protein was confirmed with Western blot and immunofluorescence.

Inflammation, and TNF-α in particular, causes cellular hyperexcitability by increasing resting membrane potential (Schäfers and Sorkin, 2008). We also find decreased expression of the K_v_4 potassium channel subunit *Kcnip2* and GABA_A_ receptor subunit *Gabrb1* in neurons supporting this development. Taken together, along with the decreased expression seen in antioxidant genes *Cat* and *Gpx3*, and increased expression of *cFos* and proinflammatory *Il6st*, these findings indicate that the CeA experiences deleterious glial-neuronal signaling during opioid withdrawal that may lead to increased and dysregulated neuronal firing. Moreover, these findings are consistent with our previous work on the effects of alcohol withdrawal on gene expression in the amygdala (Freeman et al., 2012a, b, 2013).

Unexpectedly, astrocytes demonstrated the most altered expression profiles and strongest gene expression correlation in opioid withdrawal. This is compelling in light of the finding that anxiety-like behavior elicited by microinjection of chemokines into the amygdala is mediated by astrocyte activation (Yang et al., 2016). Anxiety-like behavior in rodents and reports of severe anxiety, fear, and drug cravings in humans during opioid withdrawal is well-established (Swift and Stout, 1992; Harris and Aston-Jones, 1993). We speculate that these observations are linked—that the negative emotion experienced in opioid withdrawal is driven, in part, by neuroinflammatory glial-neuronal signaling in the CeA. Our findings, in context with the work of others, support this conjecture. For example, astrocyte TNF-α has been shown to increase AMPA receptor trafficking to the plasma membrane in neurons which may serve to strengthen anxiogenic synapses in the amygdala (Beattie et al., 2002; Coller and Hutchinson, 2012). Likewise, magnetic resonance imagining indicates anisotropy in amygdalar-specific pathways in opioid-dependent patients (Upadhyay et al., 2010). Prevalent use of anxiolytic benzodiazepine drugs in opioid dependence further implicates negative emotion in opioid withdrawal (Jones et al., 2012). We suggest astrocyte activation in the CeA during opioid withdrawal may be a key driver of these observations.

In addition, we assayed gut bacteria for all four treatments and found large differences in the relative abundance of assayed microflora in Withdrawal. The *Firmicutes* phyla, one of two major phyla that comprise the mammalian gut microbiota and an established anti-inflammatory marker, and its two major subgroups—*Clostridium coccoides* and *Clostridium leptum* had decreased abundance in Withdrawal (Figure 8). The *Butyricicoccus* genus and *Butyricicoccus pullicaecorum* also part of the *Firmicutes* phyla and anti-inflammatory, were suppressed in Withdrawal as well (Eeckhaut et al., 2013). Thus, suppression of *Firmicutes* in opioid withdrawal is observed at the phylum, subgroup, and species level. The anti-inflammatory *Bifidobacterium* genus and *F. prausnitzii* were also suppressed in Withdrawal (Sokol et al., 2008; O’Callaghan and van Sinderen, 2016). *Bacteroides*, the second major phyla of the human gut microbiota along with *Firmicutes*, and its major subgroups—*Bacteroides fragilis*, *B. vulgatus*, and *B. thetaiotaomicron*—were significantly increased in opioid withdrawal (Figure 8). Thus we present here for the first time to our knowledge evidence that opioid withdrawal decreases the *Firmicutes* to *Bacteroides* ratio: an established marker of gut microflora dysbiosis (Tamboli et al., 2004; Collins, 2014; Sampson et al., 2016; Rowin et al., 2017).

It is unclear how the gut microflora changes observed here influence opioid withdrawal syndrome or vice versa. Opioid withdrawal syndrome involves severe nausea and diarrhea, and the withdrawn rats had diarrhea and decreased food intake likely altering gut microflora. Moreover, an interoceptive circuit connecting the gut to the nucleus tractus solitarius (NTS) via the vagus nerve has been demonstrated to convey the state of the gut to the limbic system (Figure 9; Maniscalco and Rinaman, 2018). Afferents are the focus here though top-down efferents are likely involved. The NTS has strong bidirectional connections to the CeA, and we speculate that these simultaneous observations may be linked. That is, the effect exogenous opioids have on the gut via endogenous gut opioid receptors shifts gut microflora, especially in opioid withdrawal, such that the negative emotion experienced in opioid withdrawal syndrome is compounded. We are not the first to suggest gut dysbiosis may be contributing to negative reinforcement in substance dependence (de Timary et al., 2015; Skosnik and Cortes-Briones, 2016).

Negative reinforcement models posit that avoidance of unpleasant physical and emotional withdrawal symptoms motivates substance dependence. Our findings are consistent with this model. Increased activity in the amygdala can lead to negative emotion and autonomic dysregulation—both of which are associated with opioid and alcohol withdrawal and may drive drug-seeking (Tovote et al., 2015).

## Data Availability

All datasets generated for this study are included in the manuscript and/or the Supplementary Files. Both raw Ct and −ΔΔCt values of samples passing quality control are included as supplemental text files (Supplementary Tables S9, S10).

## Ethics Statement

This study was carried out in accordance with the recommendations of Animal Care and Use Committee (IACUC) of Thomas Jefferson University and Drexel University College of Medicine. The protocol was approved by Thomas Jefferson University and Drexel University College of Medicine IACUC.

## Author Contributions

SO’S performed molecular biology experiments including sample collection and microfluidic qPCR, data analysis, figure generation, and writing of the manuscript. EM supported in sample collection and laser capture microdissection. JP supported with microfluidic qPCR and data analysis. AS supported with Western blot and confocal experiments. BR supported with sample collection. JG supported with data analysis. RV, EVB, and JS designed the study and were involved with analysis, figure design, and editing. All authors discussed the results and commented on the manuscript.

## Conflict of Interest Statement

The authors declare that the research was conducted in the absence of any commercial or financial relationships that could be construed as a potential conflict of interest.

## Supplementary Material

FIGURE S1 Cell type marker expression is increased in samples of that cell type. Bar plot displays median −ΔΔCt expression values. Neurons are purple, microglia are yellow, astrocytes are green. Error bars show standard error. ^*^*p* < 0.05, ^∗∗∗^*p* < 0.0003. Tukey’s honest significance test.

FIGURE S2 Principle Component Analyses. All plots show principle component (PC) 2 on the x-axis and PC 3 on the y-axis. Circles are 10-cell pooled samples and diamonds are centroids. See Supplementary Table S3 for centroid analysis. **(A)** All samples. A distance of 1.65 was found between Placebo and Morphine centroid points, 6.90 between Placebo and Withdrawal centroid points, and 5.50 between Morphine and Withdrawal centroid points. **(B)** Plots are grouped by cell type. In neurons, a distance of 1.00 was found between Placebo and Morphine centroids, 6.40 between Placebo and Withdrawal centroids, and 5.42 between Morphine and Withdrawal centroids. In microglia, a distance 2.43 was found between Placebo and Morphine centroids, 8.76 between Placebo and Withdrawal centroids, and 7.72 between Morphine and Withdrawal centroids. In astrocytes, a distance of 1.25 was found between Placebo and Morphine centroids, 4.48 between Placebo and Withdrawal centroids, and 3.30 between Moiphine and Withdrawal centroids. **(C)** Plots are grouped by treatment. No centroid analysis performed.

FIGURE S3 Bar plots of select genes demonstrating significant differential gene expression. Error bars are standard error. Statistics were calculated using nested ANOVA (^#^*p* < 0.1, ^∗^*p* < 0.05, ^∗∗^*p* < 0.01, ^∗∗∗^*p* < 0.0001 n = 4 animals for all treatments).

FIGURE S4 TNF-α Western blot. Western blot of central nucleus of the amygdala tissue shows increased TNF-α protein in Withdrawal treatment.

FIGURE S5 Immunofluorenscent evaluation of opiold withdrawal-induced Inflammatory response 20x. Representative images of TNF-α reactivity in the CeA (n = 4 each condition and cell type). Red scale in lower right comer is 20 μm 4′-6-diamidino-2-phenylindole (DAPI) stained nuclei blue. TNF-α stained in red. **(A to D)** Neurons are stained green with neuronal nuclear antigen (NenN). **(E to H)** Microglia are stained green with CD11β). Panel **(F)** displays moderate TNF-α staining in the CeA and outside of the CeA in the lower right corner are microglia outside of the amygdala that were not surrounded by TNF-α staining. **(I to L)** Astrocytes ate stained green with glial fibrillary acidic protein (GFAP).

FIGURE S6 Immunofluorenscent evaluation of opioicl withdrawal-induced inflammatory response zoom-in. Representative images of TNF-α reactivity in the CeA (n = 4 each condition and cell type). 4′-6-diamidino-2-pheuylindole (DAPI) stained nuclei blue. TNF-α stained in red **(A to D)** Neurons are stained green with neuronal nuclear antigen (NeuN). **(E to H)** Microglia ate stauied green with CD 11β). **(I to L)** Astrocytes are stained green with glial fibiillary acidic protein (GFAP).

TABLE S1 Gene transcript primers.

TABLE S2 Heat map gene clusters.

TABLE S3 PCA and LDA centroids and distances.

TABLE S4 Principle component composite scores.

TABLE S5 Gene network edge comparisons.

TABLE S6 Nested ANOVA *p*-values.

TABLE S7 Gut microbe primers.

TABLE S8 Microbe abundance *p*-values.

TABLE S9 Raw Ct brain cell expression data.

TABLE S10 Normalized brain cell gene expression data.
