# Supplementary figures and images for: Single-Cell Glia and Neuron Gene Expression in the Central Amygdala in Opioid Withdrawal Suggests Inflammation With Correlated Gut Dysbiosis
Source: Front Neurosci. 2019 Jul 3;13:665. doi: 10.3389/fnins.2019.00665 (PMC6619439; doi:10.3389/fnins.2019.00665)

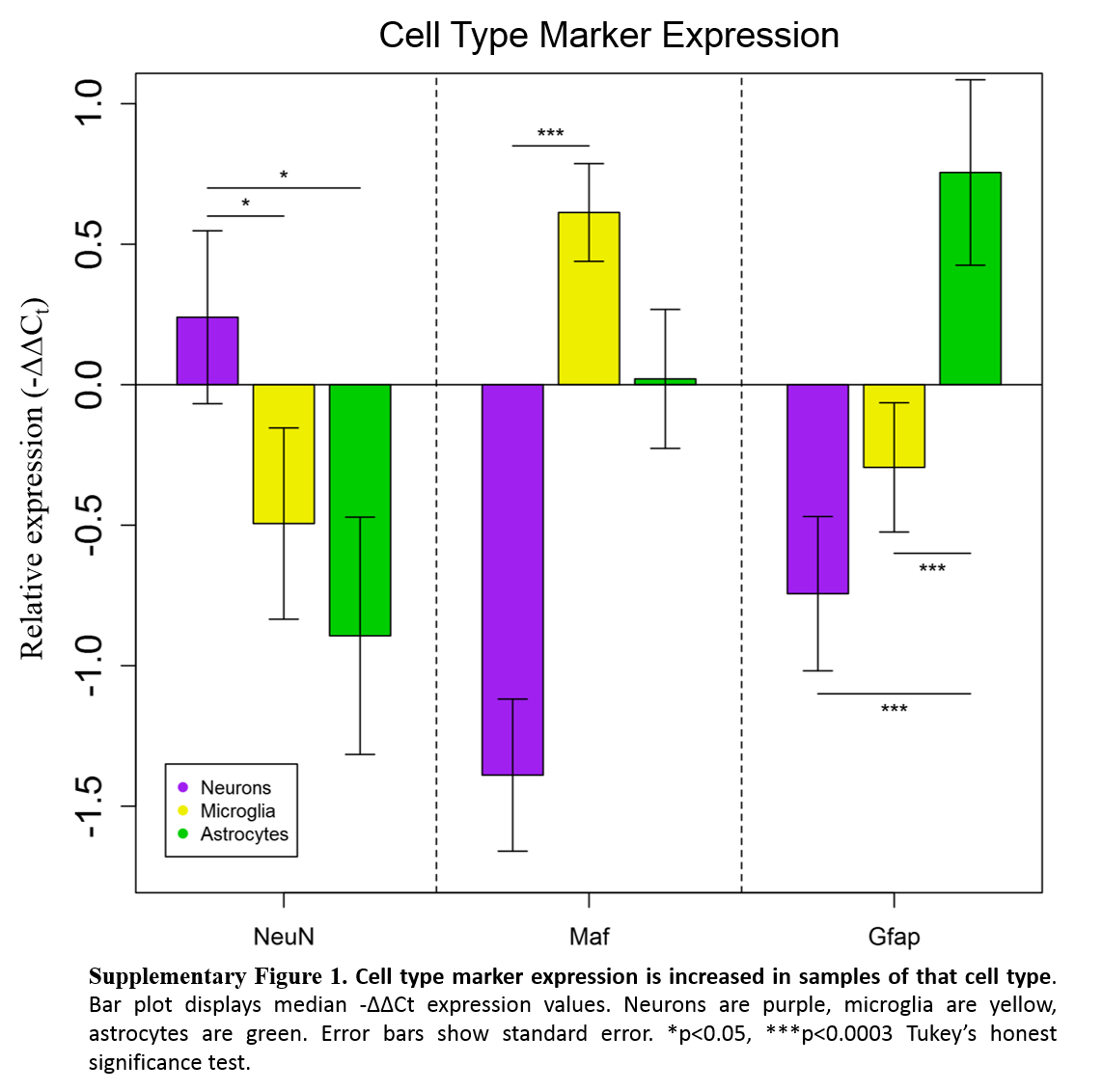

Supplement: FIGURE S1 — Cell type marker expression is increased in samples of that cell type. Bar plot displays median −ΔΔCt expression values. Neurons are purple, microglia are yellow, astrocytes are green. Error bars show standard error. *p < 0.05, ∗∗∗p < 0.0003. Tukey’s honest significance test. [file Image_1.TIF]

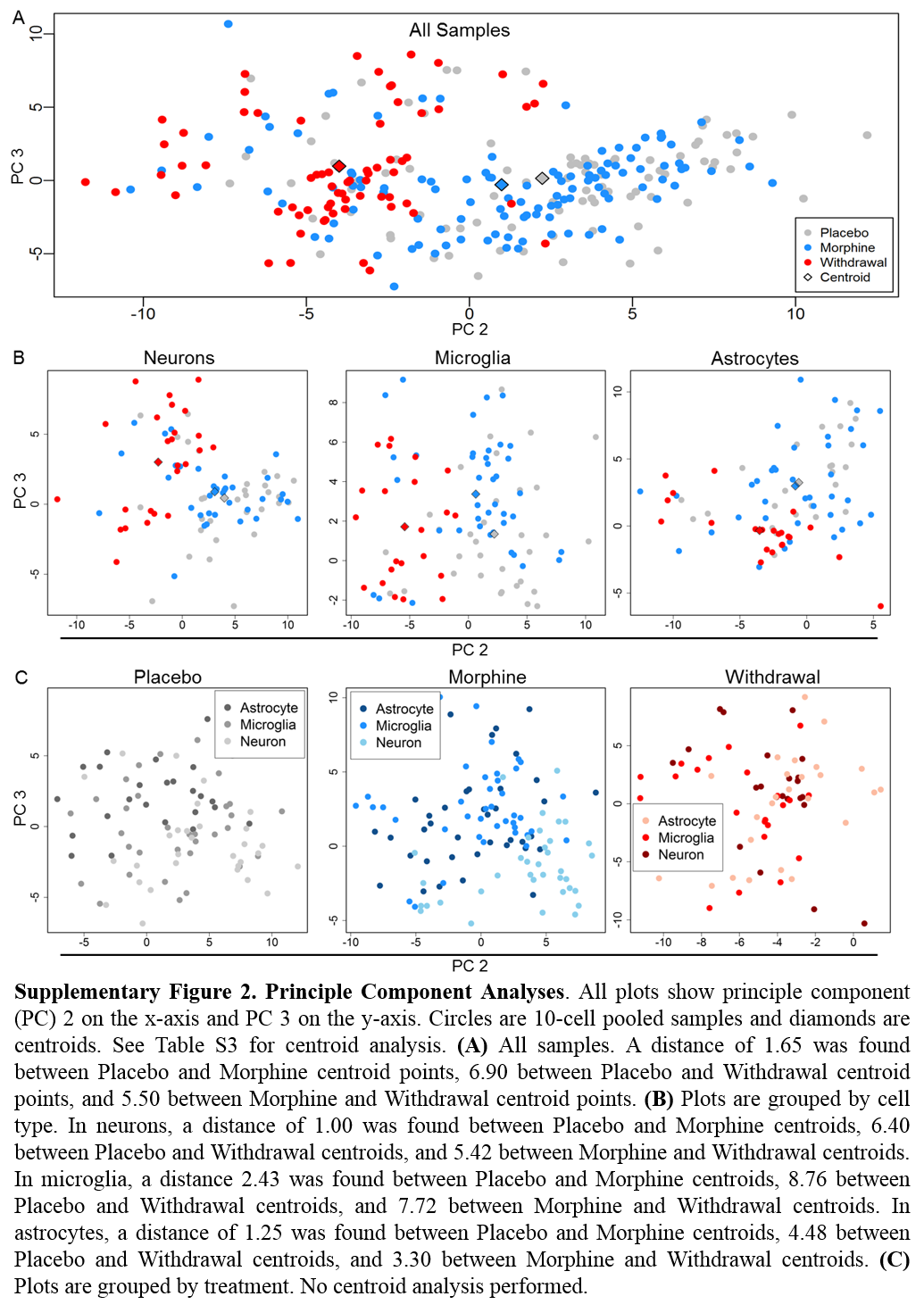

Supplement: FIGURE S2 — Principle Component Analyses. All plots show principle component (PC) 2 on the x-axis and PC 3 on the y-axis. Circles are 10-cell pooled samples and diamonds are centroids. See Supplementary Table S3 for centroid analysis. (A) All samples. A distance of 1.65 was found between Placebo and Morphine centroid points, 6.90 between Placebo and Withdrawal centroid points, and 5.50 between Morphine and Withdrawal centroid points. (B) Plots are grouped by cell type. In neurons, a distance of 1.00 was found between Placebo and Morphine centroids, 6.40 between Placebo and Withdrawal centroids, and 5.42 between Morphine and Withdrawal centroids. In microglia, a distance 2.43 was found between Placebo and Morphine centroids, 8.76 between Placebo and Withdrawal centroids, and 7.72 between Morphine and Withdrawal centroids. In astrocytes, a distance of 1.25 was found between Placebo and Morphine centroids, 4.48 between Placebo and Withdrawal centroids, and 3.30 between Moiphine and Withdrawal centroids. (C) Plots are grouped by treatment. No centroid analysis performed. [file Image_2.TIF]

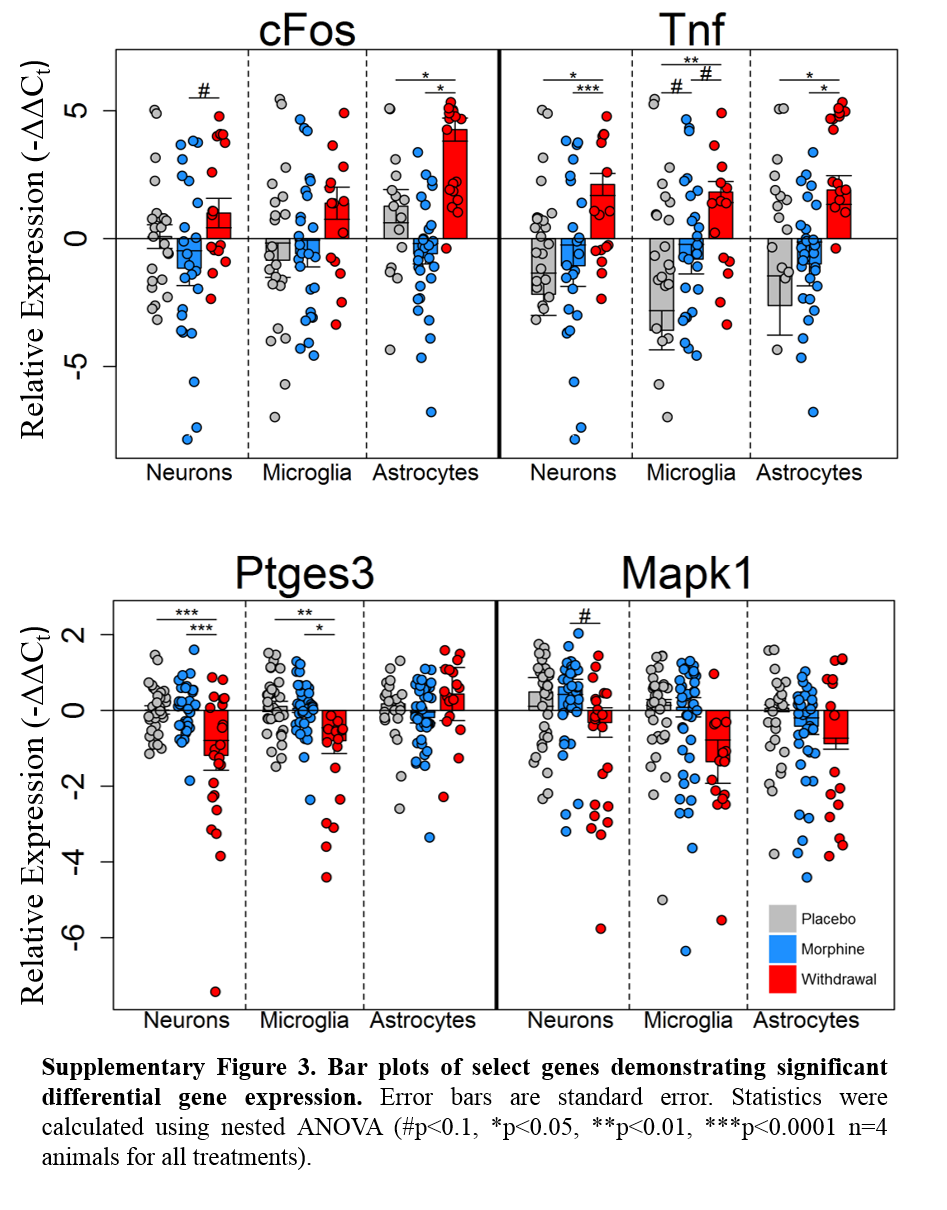

Supplement: FIGURE S3 — Bar plots of select genes demonstrating significant differential gene expression. Error bars are standard error. Statistics were calculated using nested ANOVA (#p < 0.1, ∗p < 0.05, ∗∗p < 0.01, ∗∗∗p < 0.0001 n = 4 animals for all treatments). [file Image_3.TIF]

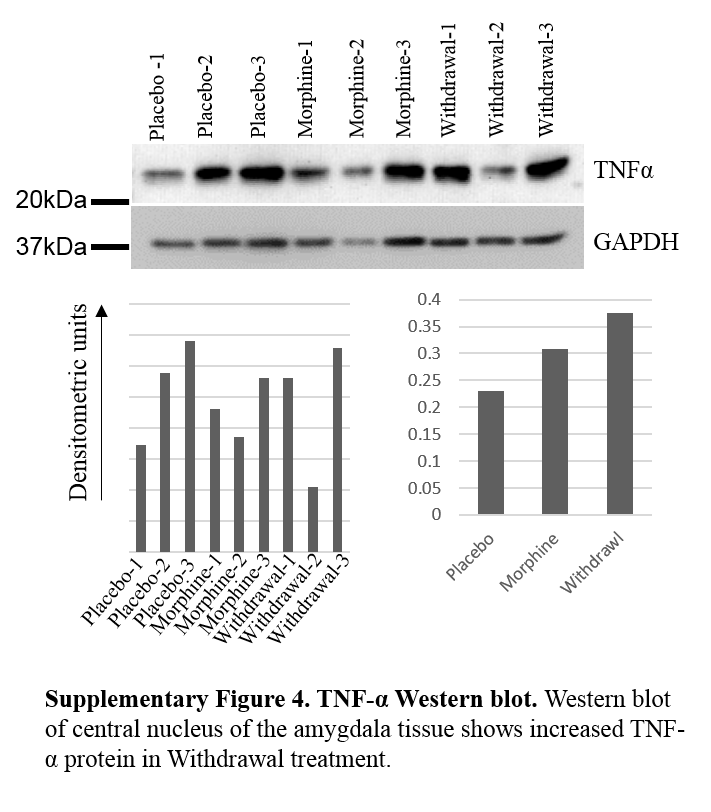

Supplement: FIGURE S4 — TNF-α Western blot. Western blot of central nucleus of the amygdala tissue shows increased TNF-α protein in Withdrawal treatment. [file Image_4.TIF]

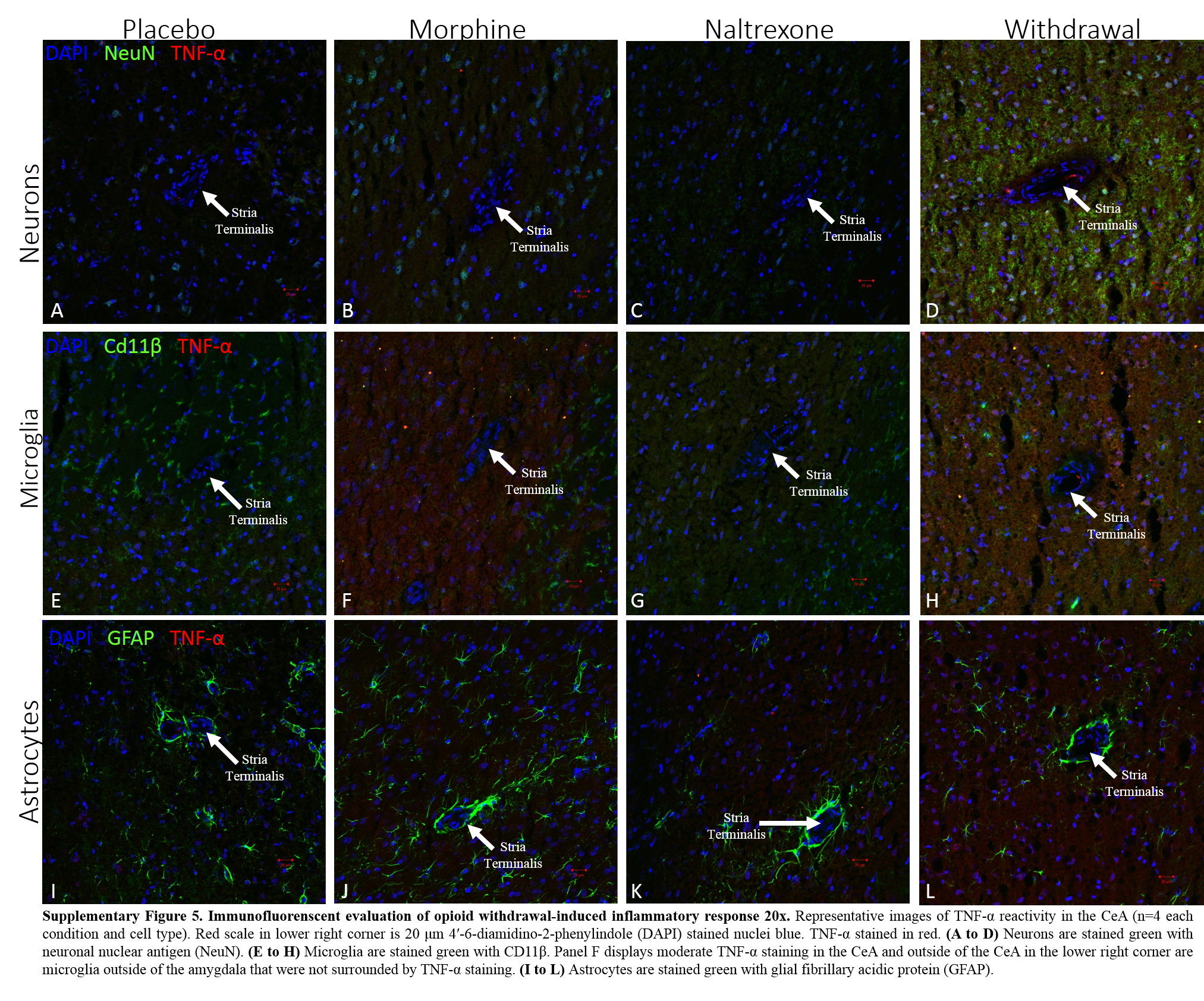

Supplement: FIGURE S5 — Immunofluorenscent evaluation of opiold withdrawal-induced Inflammatory response 20x. Representative images of TNF-α reactivity in the CeA (n = 4 each condition and cell type). Red scale in lower right comer is 20 μm 4′-6-diamidino-2-phenylindole (DAPI) stained nuclei blue. TNF-α stained in red. (A to D) Neurons are stained green with neuronal nuclear antigen (NenN). (E to H) Microglia are stained green with CD11β). Panel (F) displays moderate TNF-α staining in the CeA and outside of the CeA in the lower right corner are microglia outside of the amygdala that were not surrounded by TNF-α staining. (I to L) Astrocytes ate stained green with glial fibrillary acidic protein (GFAP). [file Image_5.TIF]

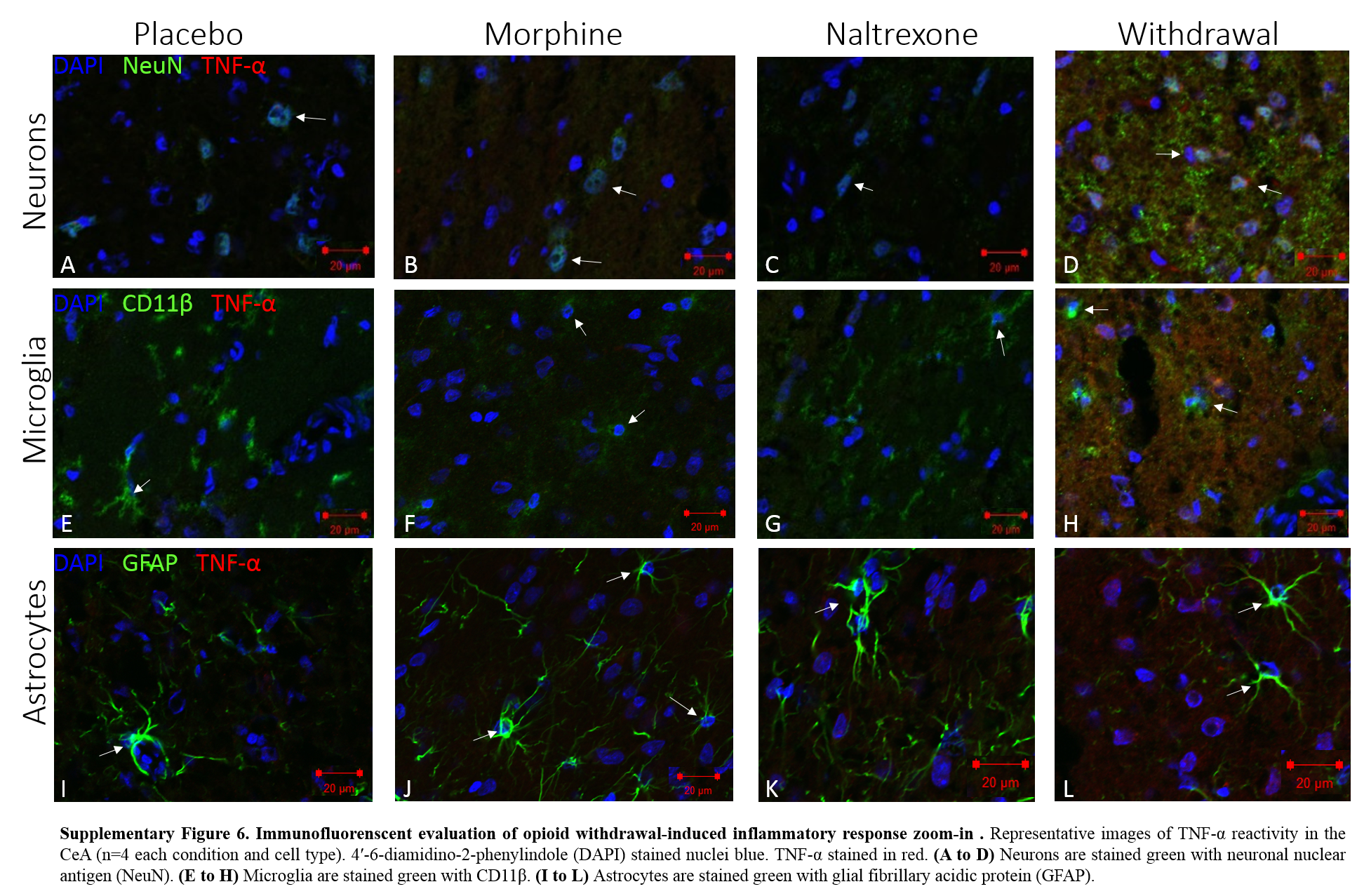

Supplement: FIGURE S6 — Immunofluorenscent evaluation of opioicl withdrawal-induced inflammatory response zoom-in. Representative images of TNF-α reactivity in the CeA (n = 4 each condition and cell type). 4′-6-diamidino-2-pheuylindole (DAPI) stained nuclei blue. TNF-α stained in red (A to D) Neurons are stained green with neuronal nuclear antigen (NeuN). (E to H) Microglia ate stauied green with CD 11β). (I to L) Astrocytes are stained green with glial fibiillary acidic protein (GFAP). [file Image_6.TIF]
